# Bottom pressure scaling of vibro-fluidized granular matter

**DOI:** 10.1038/srep17279

**Published:** 2015-11-25

**Authors:** Hiroaki Katsuragi

**Affiliations:** 1 Department of Earth and Environmental Sciences, Nagoya University, Furocho, Chikusa, Nagoya 464-8601, Japan

## Abstract

Vibrated granular beds show various interesting phenomena such as convection, segregation, and so on. However, its fundamental physical properties (e.g., internal pressure structure) have not yet been understood well. Thus, in this study, the bottom wall pressure in a vertically vibrated granular column is experimentally measured and used to reveal the nature of granular fluidization. The scaling method allows us to elucidate the fluidization (softening) degree of a vibrated granular column. The peak value of the bottom pressure *p_m_* is scaled as 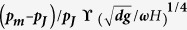
Γ, where *p*_*J*_, *d*, *g*, *ω*, *H*, and Γ are the Janssen pressure, grain diameter, gravitational acceleration, angular frequency, height of the column, and dimensionless vibrational acceleration, respectively. This scaling implies that the pressure of vibrated granular matter is quite different from the classical pressure forms: static and dynamic pressures. This scaling represents the importance of geometric factors for discussing the behavior of vibro-fluidized granular matter. The scaling is also useful to evaluate the dissipation degree in vibro-fluidized granular matter.

Vibrated granular matters show various phenomena such as convection^1^,^2^, granular Leidenfrost^3^, undulation^4^, and size segregation^5^. Some of them can also be observed in fluids, but others are not. In spite of the rich variety of phenomena, the fundamental understanding of vibrated granular matter has not yet been sufficient^6^. For example, the pressure variation within a vibrated granular matter has not been revealed while it must be a key to characterize its rheological property. In vibrated granular matters, constituent grains are driven by the vibration and gravity. These grains collectively move and exert force on the container wall. Thus, the wall pressure must be related to the vibration speed and displacement. Nevertheless, the detailed relation between them has not yet been revealed.

Physics of vibrated granular matter relates to the dynamics of planetary surface terrains. For instance, the vibration-induced fluidization of grains on the surface of small-asteroids has been considered to explain their peculiar surface terrains^7^,^8^,^9^. Some experimental works concerning this phenomenon have been performed recently^10^,^11^,^12^. However, most of them have studied the kinematics (velocity or timescale) rather than the dynamics including the pressure behavior. Further understanding of the pressure behavior would be helpful to reveal the dynamics of planetary terrains^6^.

In this study, we are going to focus on the pressure in a vibrated granular matter to discuss its fundamental dynamics. Let us consider a simple situation in which a granular column is vertically vibrated by sinusoidal oscillation with the angular frequency *ω* and amplitude *A*. Then, the displacement *x* at time *t* is written as 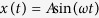. The corresponding velocity and acceleration can be written as 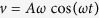 and 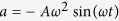, respectively. These quantities should be related to the bottom pressure *p* in the vibrated granular column of height *H*.

There are two well-known forms for the pressure scaling. When the bottom pressure is dominated by the dynamic pressure owing to the vibration, the pressure should be scaled as 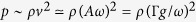, where *ρ*, *g*, and Γ denote the bulk density of granular matter, gravitational acceleration, and dimensionless maximum acceleration Γ = *Aω*^2^/*g*, respectively. In the static limit, on the other hand, the hydrostatic pressure predicts the simple scaling, 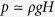. These are the two extreme situations of the pressure behavior. In this study, we find that these classical pressure scalings do not work well for explaining the behavior of the vibrated granular column. In what follows, we discuss how these scalings should be improved for the vibrated granular matter.

Some relevant experiments on the mechanical characterization of granular matter have been performed so far. The dynamic rheometry has been successfully applied to the granular shear flow^13^. For the vibrated granular matter, Hsu *et al.* have measured the relation between the vibration frequency and force applied to the container wall^14^. They defined the dynamic effective mass and found its particular behavior in very high frequency regime. Umbanhowar and van Hecke also carried out the similar characterization for the mildly vibrated granular column^15^. Recently, the flow curve of the weakly vibrated granular matter has also been measured by applying the dynamic rheometry method^16^,^17^. Although this flow curve study is the important milestone for the rheological characterization of the vibrated granular matter, their focus was limited to the weakly vibrated regime (Γ ≤ 1). In this study, we focus on the relatively low-frequency and large-acceleration regime in which the vibrated granular matter can be completely fluidized. Under such conditions, granular rheological property might become quite different from the abovementioned cases. Indeed, we find a peculiar pressure scaling form, as discussed in this paper.

In the experiment, a cylindrical granular column of diameter *D*, height *H*, and grain size *d* is shaken with frequency *f* = *ω*/2*π* and strength Γ (0 < Γ ≲ 10). Then, the vibrational acceleration *a* and bottom wall pressure *p* are measured. Figure 1(a) shows a schematic image of the experimental apparatus.

## Results

**Figure 1:**
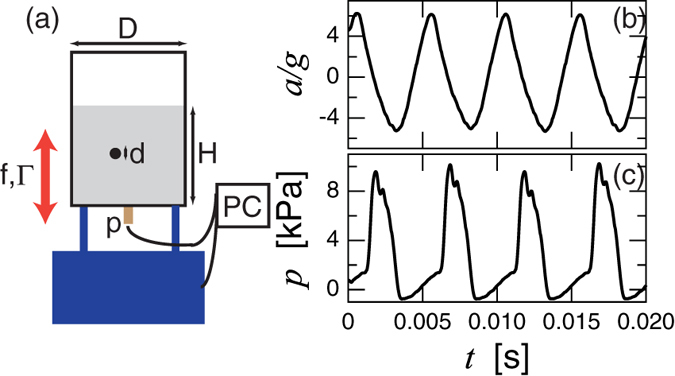
Experimental apparatus and raw data. Left panel (a) shows a schematic image of the experimental apparatus. A cylindrical container of diameter *D* is vertically shaken by an electromagnetic shaker with the frequency *f* and dimensionless maximum acceleration Γ (amplitude *A*). The column height *H* and grain size *d* are varied in the experiment. The normalized acceleration *a*/*g* and bottom pressure *p* are measured. Raw data examples are shown in (**b**) *a*/*g* and (**c**) *p*. The experimental conditions are *f* = 200 Hz, *H* = 100 mm, *D* = 75 mm, *d* = 0.8 mm, and Γ = 6.2.

A set of raw data examples of the measured *a*/*g* and *p* is shown in Fig. 1(b,c). Clear oscillation of the container can be confirmed in Fig. 1(b). Basically, the acceleration curve is smooth. It is difficult to observe any signal of the impact between the granular column and bottom wall in the raw data of *a*/*g*. In contrast, *p* shows a significantly distorted oscillation which indicates the impact of the granular column and container. Namely, the impact inertia is too weak to affect *a*/*g* signal while it is detectable by the bottom pressure sensor. By the oscillational motion itself, *p* fluctuates around <±1 kPa. This is the background oscillation which can be observed in the vibration of the empty container. Since this fluctuation level is always much smaller than the signal level, we can clearly identify the impact-induced peaks whose period is identical to that of *a*/*g*. Note that the phases are slightly different between *a*/*g* and *p*. This phase lag stems from the competition between fluidization by vibration and compaction by gravity. The splitting of pressure peaks can be slightly observed in Fig. 1(c). This splitting presumably originates from the internal grains motion within the granular column. Here we neglect this effect to focus only on the entire mechanical property rather than such a higher-order internal structures. Although the complex nature like a period doubling has been observed in a thin layer of vibrated granular matter^18^,^19^,^20^, such a bifurcation cannot be clearly observed in this experiment. This is because the height of the granular column is sufficiently tall to eliminate the complexity in this experiment.

**Figure 2:**
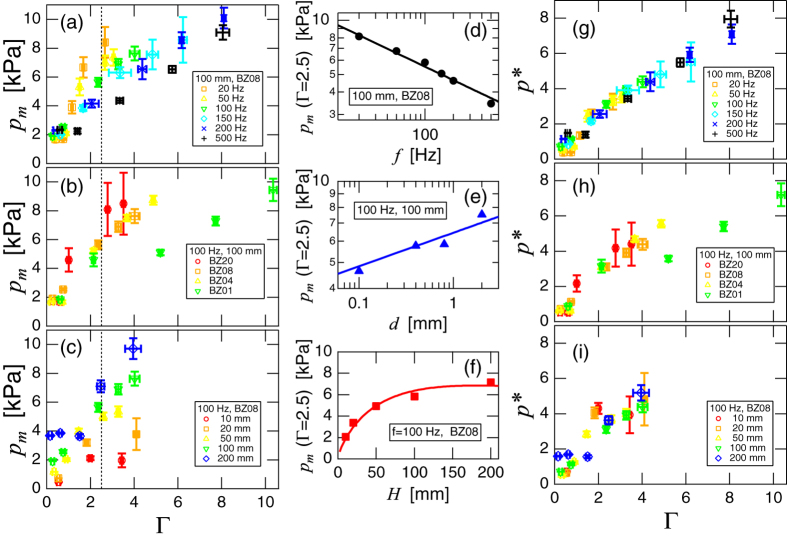
Examples of the pressure peaks and the corresponding scaling results. In the left column, *p*_*m*_ vs. Γ are plotted. The varied parameters in (**a**–**c**) are the frequency *f*, grain size *d*, and column height *H*, respectively. All the data indicate that *p*_*m*_ is the increasing function of Γ. However, the specific values depend on these parameters. To obtain scaling relations, parameter dependences are examined at Γ = 2.5 (vertical dashed lines). Plots in the middle column indicate (**d**) *p*_*m*_ ∝ *f*^−1/4^, (**e**) *p*_*m*_ ∝ *d*^1/8^, and (**f**) *p*_*m*_ ∝ *H*^−1/4^[1 − exp(−*H*/*H*_0_)]. Plots in the right column demonstrate the data collapse by the scaling 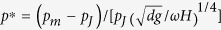. (**g**–**i**) correspond to the scaling results for (**a**–**c**), respectively. BZ01, BZ04, BZ08, and BZ20 indicate glass beads of *d* = 0.1, 0.4, 0.8, and 2 mm, respectively.

In order to characterize the vibrated granular column, we restrict ourselves to the analysis of peak amplitudes. The average peak values of acceleration *a*_*m*_ and pressure *p*_*m*_ are computed from the raw data. Parameter dependences of *p*_*m*_ are shown in Fig. 2. In Fig. 2(a–c), the data of *p*_*m*_ vs. Γ(=*a*_*m*_/*g*) are plotted. All these panels indicate that *p*_*m*_ is an increasing function of Γ. However, *p*_*m*_ depends not only on Γ but also on *f*(=*ω*/2*π*), *d*, and *H*. The goal of this analysis is to find the universal scaling law which relates *p*_*m*_ to all the relevant parameters in the system.

**Figure 3:**
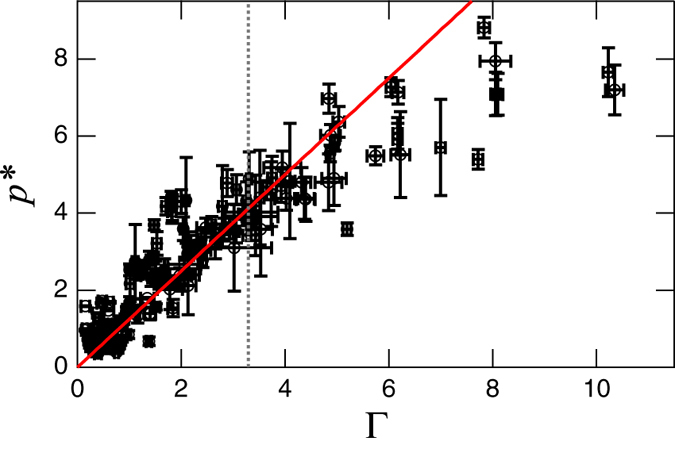
All the experimental data are scaled by the scaling 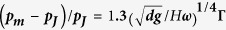 (solid red line). The vertical dashed line indicates 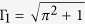.

In Fig. 2(d–f), parameter (*f*, *d*, and *H*) dependences of *p*_*m*_ at the fixed (interpolated) Γ(=2.5) are plotted. These plots demonstrate the scaling relations among *p*_*m*_, *f*, *d*, and *H*. The solid line in Fig. 2(d) indicates a relation, *p*_*m*_ ∝ *f*^−1/4^. By the least squares fitting, the value of scaling exponent is actually obtained as −0.25 ± 0.02. In Fig. 2(e), a scaling relation *p*_*m*_ ∝ *d*^1/8^ is shown as a solid line. While the estimated scaling exponent for this relation is 0.15 ± 0.03, here we presume 1/8 = 0.125 for the sake of simple dimensional analysis, as discussed below. Then, from the dimensional viewpoint, we can speculate a relation, 
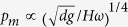
. However, *p*_*m*_ cannot be simply scaled by *H*^−1/4^. To obtain the appropriate relation, we have to consider the Janssen effect^21^ by which the pressure of the granular column is screened by side wall friction. The Janssen pressure is introduced as 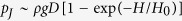, where 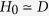 is the saturation length scale. In Fig. 2(f), the good agreement between *p*_*m*_ and *p*_*J*_*H*^−1/4^ (solid curve) is presented. Furthermore, at the static limit Γ → 0, the bottom pressure should be approximated by *p*_*J*_ rather than the hydrostatic form. By combining all the above analyses and considerations, we finally obtain a scaling relation,


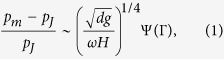


where Ψ(Γ) is a certain function. The corresponding scaling results are shown in Fig. 2(g–i). In these plots, 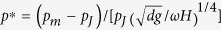 is plotted as a function of Γ. A good data collapse can be confirmed in these plots. Of course, all other data obtained in this study (but not shown in Fig. 2) can be scaled by Eq. (1). The scaling result for all data is shown in Fig. 3. One can confirm the good data collapse to a simple linear relation, i.e., Ψ(Γ) ~ Γ. Note that this experimentally obtained form is quite different from both dynamic (*p* ∝ (Γ*ω*)^2^) and static (*p* ∝ *Γ*^0^) pressure formulae. Moreover, the Janssen effect reduces the pressure value even in the vibrated granular matter.

## Discussion

In the large Γ regime, data points deviate from the linear scaling. The deviation probably results from the strong fluidization (softening) of the vibrated granular column. This behavior might relate to the inelastic bouncing ball model (IBBM)^20^,^22^. The IBBM is a simple model for a completely inelastic ball bouncing on an oscillatory base. It experiences the free fall when Γ > 1. According to the IBBM, the free-fall duration and the oscillatory period coincides at 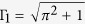. This characteristic vibration strength Γ_1_ is shown by the vertical dashed line in Fig. 3. Note that, however, the vibrated granular column is not very similar to the IBBM. The granular column must slightly dilate during its free fall. The dilation rate of the granular column could be faster than its free fall rate in order to achieve the observed steady oscillation. The oscillatory periods of *p* and *a*/*g* are identical even in the large Γ regime (e.g., the example shown in Fig. 1 corresponds to Γ = 6.2 > Γ_1_). The IBBM cannot reproduce such a steady state in Γ > Γ_1_. Whereas the effect of intersticial gas is neglected in this study, it might play a role in the vibrated granular behavior^23^,^24^,^25^. Much more detail evaluation of the interstitial-air effect is one of the most important future problems.

Let us discuss the physical meaning of the obtained scaling relation (Eq. (1)). The vibrated granular column is partially fluidized in Γ ≤ Γ_1_. In this regime, the dimensionless pressure is expressed as 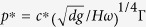 with a numerical factor c^*^ = 1.3, implying linear relation between p^*^ and Γ; Ψ(Γ) = c^*^Γ. However, the physical meaning of a scaling factor 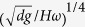 remains unclear. For a tall column, the Janssen pressure can be approximated by 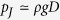. Then, using the definition Γ = *Aω*^2^/*g*, Eq. (1) can be rewritten as,


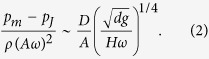


In Eq. (2), the normalized pressure is scaled by a dimensionless number 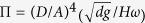; 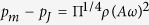. Actually, the dynamic pressure *ρ*(*Aω*)^2^ can also be regarded as a kinetic energy density per unit volume. Therefore, Eq. (2) represents a certain type of energy density balance. For the energy-based characterization of vibrated granular matter, shaking parameter *S* = (*Aω*)^2^/*dg* is usually more useful than^2^,^26^,^27^ Γ. Using the shaking parameter *S*, one can obtain a relation 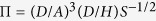 for a tall granular column. This scaling form implies that the pressure behavior is very sensitive to the geometric factor *D*/*A*. Its effect is much more crucial than the shaking strength *S*. Note that the geometric factor *D*/*A* involves both the system size and vibration amplitude. For a shallow 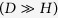 granular layer, *p*_*J*_ can be approximated by the hydrostatic pressure *ρgH*. Then, the simpler relation, 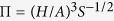, is obtained. In this case, the geometric factor *H*/*A* is the most important parameter.

The fundamental dimensionless numbers governing this system can be derived based on the above relation. There are seven independent quantities [*p*_*m*_, *p*_*J*_, *A*, *ω*, *d*, *H*, and *g*] in the system considered to obtain Eq. (1). The number of fundamental dimensions in this system is three [mass, length, and time]. Thus, in terms of the systematical dimensional analysis, there must be four independent dimensionless numbers. From Eq. (1), a set of four relevant dimensionless numbers [(*p*_*m*_ − *p*_*J*_)/*p*_*J*_, 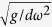, *d*/*H*, and Γ = *Aω*^2^/*g*] can be derived. In Eq. (2), two additional quantities [*ρ* and *D*] are also considered. Then, two dimensionless numbers [(*p*_*m*_ − *p*_*J*_)/*ρ*(*Aω*)^2^ and *D*/*A*] should be added in the list of dimensionless numbers. However, *ρ* and *D* are almost constant in this experiment. This implies that the form of Eq. (2) is a little more speculative than Eq. (1). The form of Eq. (2) is introduced to discuss the underlying physics in the vibro-fluidized granular matter. In addition, the effect of packing fraction is neglected in these forms since the packing fraction is directly related to *ρ* and Γ^28^, i.e., the packing fraction is not an independent quantity. Moreover, the value of packing fraction in this study is almost constant.

The obtained scaling form can also be compared with previously obtained energy dissipation scaling^29^,^30^,^31^,^32^. Equation (2) corresponds to the energy scaling 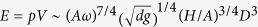, where *V* ~ *D*^2^*H* and *E* are the volume and internal energy of the system, respectively. Here, we assume that the dilation of the vibrated granular column is negligible (*V* = *const.*) for the internal energy estimate. Thus, Eq. (2) implies *E* ∝ (*Aω*)^*α*^ with *α* = 7/4 = 1.75. This type of internal energy (dissipation) scaling has been discussed in some previous studies^29^,^30^,^31^,^32^. In these previous works, *α* distributes around 1.5. Although the current experimental value is slightly larger than 1.5, it is less than the theoretically obtained value^30^ *α* = 2. Since the dissipation due to the internal viscosity or friction is not considered in the current scaling analysis, the gravitational velocity 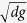 plays an essential role to ensure the dimensionally sound relation. To improve the scaling form, we must explicitly take into account the dissipation effects. Such an improvement is open to future.

Since the obtained scaling form is written in a dimensionless form, it can be applied to various scale phenomena. However, we must be careful in specific applications. First, the parameter ranges varied in this experiment are not very wide (approximately one order of magnitude; see Methods section for details). Second, some more additional factors have to be considered to completely understand the rheological property of the vibrated granular matter. As mentioned before, interstitial-air effect is neglected in this study. In addition, the phase lag between *p* and *a*/*g* can be a useful information for the rheological characterization of the vibrated granular matter. We have tried to compute the phase lag from the cross-correlation peak of *p* and *a*/*g*. However, the data are very noisy and it was difficult to extract the meaningful information from the phase lag data. The precise measurement of the phase lag and its characterization are important future issues.

In summary, the degree of fluidization in a vibrated granular column was characterized by measuring the vibrational acceleration and the bottom pressure. From the experimentally obtained data, we found a scaling for the bottom pressure (Eq. (1)). This scaling is quite different from both the dynamic and static pressure forms. By the scaling analysis, the importance of geometric factors to discuss the vibro-fluidized granular pressure was revealed. Besides, the obtained scaling is somewhat similar to the previously discussed internal energy scaling form whereas the dissipation effect is not explicitly considered in this study. That is, the scaling partially elucidated the complex rheological property of the vibrated granular column.

## Methods

We build a simple experimental apparatus as shown in Fig. 1(a). A cylindrical container of inner diameter *D* = 75 mm is mounted on an electromagnetic shaker (EMIC 513-B/A). At the bottom of the container, a pressure sensor (KYOWA electronic instruments Co., Ltd., PGM-02KG) is fixed to measure the bottom pressure *p*. This system is almost identical to that used in previous studies of the plunged granular column^33^,^34^. Glass beads are poured into the container. The height of the column *H* is ranged from 10 to 200 mm. The diameter of glass beads *d* is varied from 0.1 to 2 mm (roughly monodisperse). The vibrational acceleration is measured by an accelerometer (EMIC 710-C) which is mounted on the wall of the container. The range of Γ (vibration strength) in this experiment is 0 < Γ ≲ 10. The vibration frequency *f* = *ω*/2*π* is varied (independent of Γ) in the range of 20 ≤ *f* ≤ 500 Hz. The data are taken for 30 s for each experimental condition. Approximately one minute pre-vibration is applied to achieve the steady vibration before each measurement. All the experiments are carried out under the atmospheric condition (not vacuum). The variation ranges of these parameters are about factor of 20 (slightly over one order of magnitude).

The vibrated granular column seems to be well-fluidized in this experiment. In the strongly vibrated granular column, various phenomena can be observed^27^. In this experiment, the upper limit of Γ is approximately 10 (much smaller than experiments by Eshuis *et al.*^27^ but within the fluidized regime). When Γ is approximately larger than unity, the granular convection is observed as reported by Yamada and Katsuragi^11^. Previous experiments on the dynamic Janssen effect and mildly vibrated granular column have used the total effective mass rather than the local bottom wall pressure^15^,^35^. However, here we measure the bottom wall pressure of the vibrated granular column since such a setup is more convenient to characterize the rheological property^14^. Although we neglect the interstitial-air effect on the basis of the former experimental result^36^, the interstitial-air effect might be serious particularly for small grains of *d* = 0.1 mm. In the analysis, dissipation effects such as air viscosity and wall friction have not been explicitly considered.

## Additional Information

**How to cite this article**: Katsuragi, H. Bottom pressure scaling of vibro-fluidized granular matter. *Sci. Rep.* **5**, 17279; doi: 10.1038/srep17279 (2015).
